# A Systematic Follow-Up of *Mycobacterium tuberculosis* Drug-Resistance and Associated Genotypic Lineages in the French Departments of the Americas over a Seventeen-Year Period

**DOI:** 10.1155/2014/689852

**Published:** 2014-03-13

**Authors:** Julie Millet, Elisabeth Streit, Mylène Berchel, Anne-Gaël Bomer, Franziska Schuster, Delaina Paasch, Jessica Vanhomwegen, Gilbert Cadelis, Nalin Rastogi

**Affiliations:** ^1^WHO Supranational TB Reference Laboratory, Unité de la Tuberculose et des Mycobactéries, Institut Pasteur de Guadeloupe, 97183 Abymes, Guadeloupe, France; ^2^Service de Pneumologie, CHU de Pointe-à-Pitre, 97110 Pointe-à-Pitre, Guadeloupe, France; ^3^Centre de Lutte Antituberculeux de la Guadeloupe, CHU de Pointe-à-Pitre, 97110 Pointe-à-Pitre, Guadeloupe, France

## Abstract

The population of the French Departments of the Americas (FDA) is highly influenced by the intense migratory flows with mainland France and surrounding countries of the Caribbean and Latin America, some of which have high incidence rates of tuberculosis (Haiti: 230/100,000; Guyana: 111/100,000; and Suriname: 145/100,000) and drug resistance. Since the development of drug resistance to conventional antituberculous drugs has a major impact on the treatment success of tuberculosis, we therefore decided to review carefully *Mycobacterium tuberculosis* drug resistance and associated genotypic lineages in the FDA over a seventeen-year period (January 1995–December 2011). A total of 1239 cases were studied, including 153 drug-resistant and 26 multidrug-resistant- (MDR-) TB cases, representing 12.3% and 2.1% of the TB cases in our study setting. A significantly higher proportion of *M. tuberculosis* isolates among relapse cases showed drug resistance to isoniazid (22.5%, *P* = 0.002), rifampicin (20.0%, *P* < 0.001), or both (MDR-TB, 17.5%; *P* < 0.001). Determination of spoligotyping based phylogenetic clades showed that among the five major lineages observed—T family (30.1%); Latin-American and Mediterranean (LAM, 23.7%); Haarlem (H, 22.2%); East-African Indian (EAI, 7.2%); and X family (6.5%)—two lineages, X and LAM, were overrepresented in drug-resistant and MDR-TB cases, respectively. Finally, 19 predominant spoligotypes were identified for the 1239 isolates of *M. tuberculosis * in our study among which 4 were significantly associated with drug resistance corresponding to SIT20/LAM1, SIT64/LAM6, SIT45/H1, and SIT46/undefined lineage.

## 1. Introduction

Tubercle bacilli having developed resistances to conventional treatments have a major impact on the way patients are treated. While first-line antibiotics are sufficient to ensure a successful treatment of most patients infected with drug susceptible *Mycobacterium tuberculosis* strains, infections caused by so-called multiresistant (MDR) strains, defined as strains resistant to rifampin and isoniazid, necessitate the use of second-line antibiotics. These drugs are likely to cause more severe side effects and are also more costly than first-line antibiotics [1, 2]. Strategies devised to control the spread of resistant and multiresistant *M. tuberculosis* strains inevitably include systematic drug susceptibility testing of bacterial isolates and thorough follow-up of patient treatment.

The population of the French departments of the Americas (FDA; almost 1 million in 2011) is a heterogeneous mix resulting from ongoing migratory flows from metropolitan France, from neighboring countries like Suriname and Brazil as well as from more distant countries such as China for French Guiana (representing a continental setting) and Haiti, St. Lucia and Dominica for Guadeloupe and Martinique (both representing insular island settings). In such a context, imported cases of tuberculosis (TB) including drug-resistant cases are not uncommon, and the TB epidemiology in French departments is largely influenced by migration. As of 2010, the TB epidemic in the three FDA was rather heterogeneous [3]; a higher TB incidence was observed for both French Guiana and Guadeloupe (incidence of 15.6 and 9.7 cases/100,000 inhabitants, resp.), as compared to a significantly lower incidence rate observed in Martinique (3.8 cases/100,000).

Anticipating the challenge in TB control resulting from regional disparities, heterogeneous population, and migratory movements dependent TB transmission, the TB & Mycobacteria Unit of the Institut Pasteur de la Guadeloupe (IPG) was asked by the regional health authorities not only to ensure a centralized bacteriological diagnosis of all TB cases but also to systematically follow up *M. tuberculosis* drug-resistance and associated genotypic lineages in the French departments since 1994. For this purpose, we specifically developed an expertise in genotyping using spoligotyping [4] and mycobacterial interspersed repetitive units—variable number of tandem DNA repeats (MIRU-VNTRs [5]), coupled to surveillance and tracking of *M. tuberculosis* complex (MTBC) clones thanks to the establishment of in-house international genotyping databases [6, 7].

The purpose of this paper is to review TB cases caused by drug-resistant and MDR *M. tuberculosis* isolates in the French departments during a 17-year period from January 1995 to December 2011. Observed resistance profiles, circulating *M. tuberculosis* genotypes and epidemiological data on TB patients, were analyzed to ensure a thorough characterization of the cases resulting from drug-resistant isolates.

## 2. Materials and Methods

### 2.1. Bacterial Isolates and Patients

All clinical samples taken for TB diagnosis in Guadeloupe are directly sent to the mycobacteria laboratory of the IPG which also receives samples and mycobacterial cultures from Martinique and French Guiana. The present study is a descriptive, retrospective study including all patients for which a positive *Mycobacterium tuberculosis* complex (MTBC) culture was obtained in the three FDA between January 1995 and December 2011. It comprised a total of 1239 TB cases: 346 from Guadeloupe, 177 from Martinique, and 716 from French Guiana. Demographic and epidemiological information concerning the patients were collected using a disclosure form that was filled in as a part of the examination request. All strains included in this study had a complete antibiotic susceptibility profile and a spoligotyping profile and strains not fulfilling both conditions (*n* = 28) were excluded from this study. Cases were further differentiated as “new” (*n* = 1199) versus “persistent” (*n* = 40) cases; a TB case was considered persistent if positive *M. tuberculosis* cultures were obtained for the same patient at an interval of more than 6 months, and for which available spoligotyping profiles of *M. tuberculosis* isolates were identical. Regarding HIV serology, patients were not systematically tested for HIV; nonetheless, this information was available for 41.3% (*n* = 512/1239) of the patients.

### 2.2. Drug Susceptibility Testing and Genotyping

The IPG laboratory is one of the supranational TB reference laboratories (SRL-TB) of the World Health Organization (WHO) and regularly participated in the proficiency testing within the network of SRLs [8]. Drug susceptibility testing (DST) was performed using the 1% proportion method using the 7H11 agar [9, 10] or liquid medium using the Bactec MGIT960 methodology [11] to the following drugs: isoniazid (INH), rifampin (RIF), ethambutol (EMB), streptomycin (SM), and pyrazinamide (PZA). Strains displaying resistance to one or several of these drugs were classified as resistant and strains with cumulative resistance to INH and RIF were classified as multidrug resistant (MDR). MDR strains were further tested, using the 1% proportion method on 7H11 agar [9, 10] or liquid medium with the Bactec MGIT960 methodology [11] to following second-line drugs [10, 12]: kanamycin (KAN), amikacin (AMIK), capreomycin (CAP), ofloxacin (OFL), ciprofloxacin (CIP), ethionamide (ETH), and rifabutin (RBT). Note that the use of liquid versus solid medium depended on the evolution of DST methods; thus, DST was performed on solid medium before 2007, and on liquid medium after this date.

Starting in 2007, the phenotypic resistance of MDR isolates was further reconfirmed by detection of mutations to the *rpoB* and *katG* genes (and more recently also the *inhA *gene) that confer resistance to RIF (*rpoB*) and INH (*katG* and *inhA*), respectively [13], by using a commercially available line-probe assay (GenoType MTBDRplus, Hain Lifescience, Nehren, Germany). From 2011 onwards, we further added the GenoType MTBDRsl test (Hain Lifescience, Nehren, Germany), which allows detecting the most common mutations in *gyrA* gene for resistance to fluoroquinolones, in *rrs* gene for resistance to amikacin, capreomycin, and kanamycin, and in *embB* gene for ethambutol resistance, (http://www.hain-lifescience.de/en/products/microbiology/mycobacteria/genotype-mtbdrsl.html).

Note that despite the use of genetic determination of drug-resistance mutations, the culture-based drug-susceptibility testing to 1st-line drugs (and 2nd-line drugs for MDR strains) was performed systematically on all isolates so as not to miss any case(s) of phenotypic drug resistance.

Spoligotyping, a PCR based reverse hybridization method, was carried out to study the polymorphism of the Direct Repeat (DR) locus as previously described (4) on bacterial DNAs. Starting from 2007, 12-loci MIRU-VNTRs were also used as a second-line typing method [5], allowing differentiating *M. tuberculosis* strains based on the copy number of the 12 selected loci; a MIRU-VNTR profile was thus available for 661 of the strains included in this study.

### 2.3. Database Comparison and Statistical Analysis

Spoligotypes (in octal format) and MIRU-VNTRs were compared by using the SITVIT2 proprietary database of the Pasteur Institute of Guadeloupe, which is an updated in-house version of the publicly released SpolDB4 [6] and SITVITWEB [7] databases. In this database, spoligotype international type (SIT) and MIRU international type (MIT) designate an identical pattern shared by two or more patient isolates by spoligotyping and MIRU-VNTRs, respectively, whereas “orphan” designates a pattern reported for a single isolate that does not correspond to any of the patterns recorded in the database repository. Major phylogenetic clades were assigned according to rules in SITVITWEB [7]; these included specific signatures for various *M. tuberculosis* complex members as well as rules defining major lineages/sublineages for *M. tuberculosis sensu stricto*, that is, the Beijing clade, the Central Asian (CAS) clade and two sublineages, the East African-Indian (EAI) clade and nine sublineages, the Haarlem (H) clade and three sublineages, the Latin American-Mediterranean (LAM) clade and 12 sublineages, the “Manu” family and three sublineages, the S clade, the IS*6110*- low-banding X clade and four sublineages, and an ill-defined T clade with five sublineages.

Statistical analysis was carried out using Stata version 12 (Stata Corporation, College Station, TX, USA). The Chi2 test and Fisher's exact test were used to compare proportions and Student's *t*-test was used for the comparison of averages. Odd ratios have been calculated in order to evaluate possible associations of the circulating genotypes with drug resistance.

## 3. Results

### 3.1. Demographical Characteristics of Patients and Drug Resistance

The mean age of TB patients for all the 3 departments was 42.6 years (data available for 1184/1239 patients). Most of the patients (82.9%) were between 15 and 65 years old, 3.9% were younger than 15 years, and 13.2% older than 65 years. The male-to-female ratio was 1.8 (data available for 1221/1239 patients; Table 1). During the study period, 17–39 cases of drug resistance (1–7 cases of MDR-TB) were observed per year. These figures reflect a yearly range of 7.8%–18.4% for total drug resistance (including 0.5%–3.3% of MDR-TB cases). Drug-resistant cases concerned 15.2% of patients aged below 15 years. This age group was not concerned by multidrug resistance since no MDR-TB strains were observed in patients younger than 15 years (resistance profiles in this age group: SM/INH, *n* = 2; SM/PZA, *n* = 1; INH/PZA, *n* = 1; INH, *n* = 2, PZA, *n* = 1). Regarding epidemiologic data like age, sex ratio, geographic origin, or HIV serology, no significant differences could be observed when comparing patients harboring drug resistant (including MDR) strains versus patients infected by drug susceptible strains (Table 1). The majority of the cases were smear-positive, regardless of their drug susceptibility status. However, the proportion of persisters (versus new TB cases) was significantly higher when the infection was caused by a drug-resistant or MDR strain (*n* = 11/153 or 7.2% of cases with drug-resistant strains, *P* = 0.007; and *n* = 7/26 or 26.9% of cases with MDR strains, *P* < 0.001).

*M. tuberculosis* strains displaying a resistance to at least INH were responsible for 7.0% of the new cases and 22.5% of the persistent cases (*P* = 0.002). Likewise, 2.3% of the new cases and 20.0% of the persistent cases were caused by strains presenting at least a resistance to RIF (*P* < 0.001) (Table 2). The proportion of *M. tuberculosis* strains resistant to two antibiotics is also significantly higher among the persistent cases (17.5% versus 2.6% of new cases; *P* < 0.001) and the same goes for MDR cases (17.5% versus 1.6% of new cases; *P* < 0.001). Note that all cases of persistent TB caused by strains resistant to two antibiotics were due to MDR strains. For new cases with *M. tuberculosis* strains resistant to 2 antibiotics, the following combinations of drug resistances were observed: INH/SM (13/31), IHN/RIF (MDR; 10/31), SM/PZA (6/31), INH/EMB (1/31), and INH/PZA (1/31).

The rates of primary resistance and primary multiresistance (i.e., proportion of cases with *M. tuberculosis* resistance/multiresistance observed in new cases) were 11.8% (*n* = 142/1199) and 1.6% (*n* = 19/1199), respectively. Drug resistance profiles of the 19 primary MDR isolates (out of 26 MDR cases in total) showed that *n* = 6/19 displayed the following additional resistances to first-line drugs (MDR/SM, *n* = 3/19, MDR/SM/EMB, *n* = 2/19, and MDR/EMB, *n* = 1/19). The remaining 5/19 primary MDR isolates showed combined resistance to one or more second-line drugs (in 2 cases, resistance to 1st-line drugs other than INH and RIF) with the following drug-resistance patterns: MDR/ETH, MDR/ETH/CIP, MDR/RBT, MDR/RBT/SM/EMB, and MDR/EMB/PZA/CIP/OFL/RBT. Among the 7 secondary MDR isolates (all from patients with persistent TB), the following combinations of drug resistances were observed: MDR alone (*n* = 3/7), MDR/RBT (*n* = 2/7), MDR/SM (*n* = 1/7), and MDR/CIP/ETH (*n* = 1/7). Last but not the least, no case of extensively drug resistant TB (XDR-TB, which refers to MDR-TB that is also resistant to a fluoroquinolone and at least one of three injectable second-line anti-TB drugs, capreomycin, kanamycin, or amikacin) was observed during the study period. However, cases of pre-XDRTB, defined as MDR isolates displaying additional resistance to any of the fluoroquinolones or one of the injectable second-line drugs but not both, were found (*n* = 3; MDR/CIP; MDR-CIP/OFL).

Regarding genotypic resistance, MDR strains isolated in the later years of the study (*n* = 5) were also analyzed using the MTBDR*plus* assay. While all 5 strains displayed the same INH resistance conferring mutation MUT1 of the *katG* gene (mutation AGC-ACC at position 315 corresponding to S315T change in amino acid sequence), only 2 of the strains hybridized with a *rpoB* mutation probe (MUT2B (CAC-CAG; H526D) and MUT 3 (TCG-TTG; S531L), resp.). RIF resistance was rather characterized by the absence of hybridization with one or more (maximum 2) of the “wild type” probes (Table 3). Interestingly, a total of 3/5 isolates presented a rarely observed MTBDR*plus* profile characterized by an absence of hybridization of probe WT2 alone (*n* = 2) or associated with a nonhybridization of probe WT4 (*n* = 1). According to MTBDR*plus* algorithm, this absence of hybridization of probe WT2 testifies to a mutation in codons 510–512 since WT3 gave a hybridization signal in all three strains. Moreover, 2/5 isolates showed an absence of the WT4 hybridization signal, combined with the presence of both WT3 and WT5. This result is to be further investigated by sequencing since it is not considered by the MTBDR*plus* algorithm (all codons covered by WT4 being covered by either WT3 or WT5). The remaining MDR strain showed signal for WT8 but hybridized with MUT3 indicating a mutation in codon 531 (TCG-TTG; S531L).

### 3.2. Combined Analysis of Circulating Genotypes and Drug Resistance Profiles

Spoligotyping yielded 281 distinct profiles for the 1199 MTBC isolates classified as new cases. Of the 166 unique profiles observed in this study, 46 were attributed orphan status upon comparison to SITVIT2. The remaining 115 spoligotypes clustered 86.2% (*n* = 1033/1199) of the strains (2–135 strains/cluster). A significantly higher clustering rate was observed among drug susceptible strains as compared to resistant strains (*n* = 902/1057 or 85.3% versus *n* = 109/142 or 76.8% for drug-sensitive and resistant strains, resp.; *P* < 0.01).

Lineage attribution showed that T, LAM, and H were by far the most common clades, accounting for 29.9% (358/1199), 23.9% (286/1199), and 22.1% (265/1199) of the isolates, respectively. The EAI (7.1%; 85/1199) and X (6.7%; 80/1199) lineages were observed frequently while other lineages and MTBC members were of minor importance. These were Beijing (*n* = 18/1199 or 1.5%), S (*n* = 15/1199 or 1.3%), *M. africanum* (*n* = 8/1199 or 0.7%), *M. bovis* (*n* = 4/1199 or 0.3%), LAM10-CAM (*n* = 4/1199 or 0.3%), CAS (*n* = 2/1199 or 0.2%), Manu (*n* = 2/1199 or 0.2%), and LAM7-TUR (1/1199 or 0.1%). The remaining 71 strains could not be attributed a phylogenetic lineage. The LAM lineage was overrepresented among MDR strains as opposed to drug susceptible strains or strains presenting other resistance profiles (42.3% versus 23.3%; *P* = 0.02) while the X family and “undefined” genotypes are particularly frequent among strains presenting resistances other than MDR in comparison to sensitive strains (10.5% versus 6.0%; *P* = 0.04 for the X clade and 15.0% versus 9.6%; *P* = 0.04 for “undefined” genotypes) (Table 1, Supplemental Tables S1 and S2 in Supplementary Material available online at http://dx.doi.org/10.1155/2014/689852).

**Figure 1:**
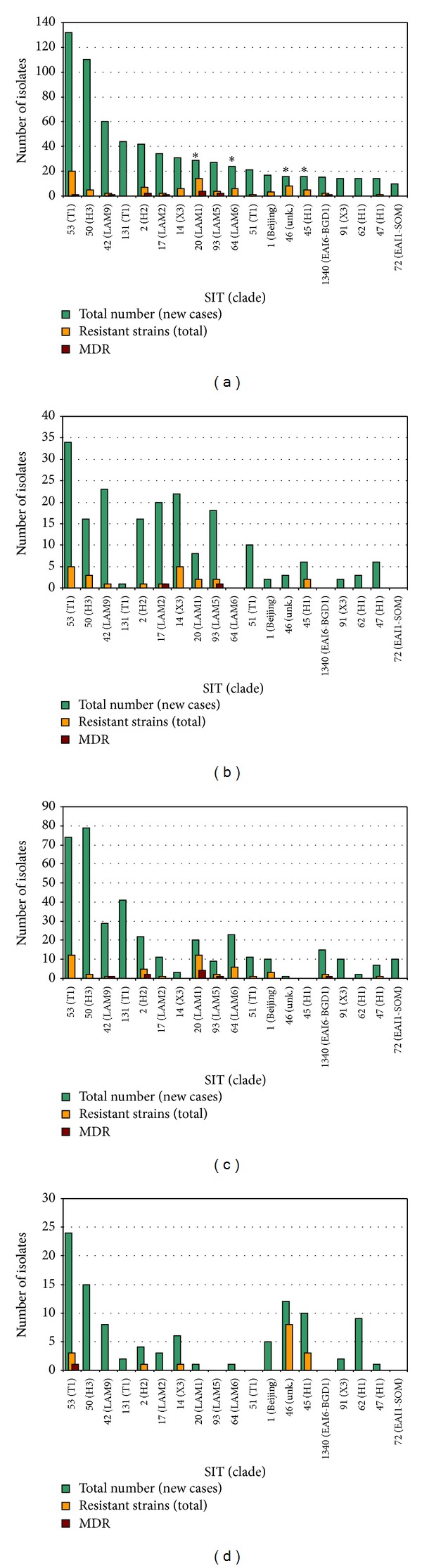
Total number of isolates, drug-resistant (includes MDR) and MDR isolates for the 19 most frequent spoligotypes (representing more than 55% of all isolates; *n* = 670/1199) in the three French Departments of Americas considering only new TB cases. (a) Total study sample; (b) Guadeloupe; (c) French Guiana; (d) Martinique. Note that asterisk denotes spoligotypes showing a significant association with resistance to first-line anti-TB drugs (odd ratio with 95% confidence interval: 7.9 (3.6–17.2); 3.8 (1.3–11.5); 8.4 (3.1–23.4); and 2.8 (1.1–7.4), resp., for SIT 20, 45, 46, and 64).

More than half of the isolates (*n* = 670/1199; 55%) were clustered by 19 major spoligotypes (Figure 1). Furthermore, 4 spoligotype profiles, namely, SIT20, SIT45, SIT46, and SIT64, were found to harbor significantly higher proportions of resistant strains than other spoligotypes in the French Departments of the Americas (48.3%, 31.3%, 50.0%, and 25.0% of drug-resistant isolates, resp.). The corresponding odd ratios, measuring association between the genotypic profiles (i.e., spoligotypes) and drug resistance, were significantly higher for these 4 spoligotype profiles than for other spoligotypes (SIT20: OR = 7.9 [3.6–17.2], *P* < 0.001; SIT45: OR = 3.8 [1.3–11.5], *P* = 0.016; SIT46: OR = 8.4 [3.1–23.4], *P* < 0.001; SIT64: OR = 2.8 [1.1–7.4], *P* = 0.035; ORs are given with 95% confidence intervals). Geographical repartition of the 19 major spoligotypes (Figures 1(b) to 1(d)) revealed their unequal distribution among the 3 French Departments. Indeed, French Guiana showed a significantly higher proportion of strains with patterns corresponding to SIT131 as compared to the 2 insular departments (SIT 131: 6% versus 0.3% in Guadeloupe, *P* < 0.001; and 6% versus 1.1% in Martinique, *P* = 0.003). Furthermore, note that SIT1340 and SIT72 (both classified among the ancestral EAI lineage) were not observed in either of the two islands—Guadeloupe and Martinique. On the other hand, a significant overrepresentation of SIT14 in Guadeloupe and Martinique in comparison to French Guiana (5.9% and 3.5% in Guadeloupe and Martinique versus 0.4% in French Guiana; *P* < 0.01) was observed. Guadeloupe was also characterized by significantly higher proportions of strains with spoligotypes corresponding to SIT17 (6.3% in Guadeloupe versus 1.7% (*P* = 0.02) and 1.6% (*P* < 0.001) in Martinique and French Guiana, resp.) and SIT93 (5.3% versus 1.3% in French Guiana, *P* = 0.002, but absent in Martinique). However, Martinique showed higher proportions of strains with SIT45 (5.7% versus 1.8% in Guadeloupe, *P* = 0.0182, absent in French Guiana), SIT46 (6.8% versus 0.9%, *P* = 0.003 in Guadeloupe, and 0.1%, *P* < 0.0001 in French Guiana), and SIT62 (5.1% versus 0.9%, *P* = 0.004 in Guadeloupe, and 0.3%, *P* < 0.001 in French Guiana).

Clusters of new cases including at least 2 drug-resistant strains of *M. tuberculosis* were studied in detail in order to identify cases of recent transmission of resistant strains. Of the 38 clusters containing resistant strains, 21 clusters (named A-U) included 2 or more cases of drug-resistant strains (*n* = 109; proportion of resistant cases between 3.3% for cluster C to 100% for clusters R and U, Table 4). Associating the spoligotypes of the 109 drug-resistant isolates grouped in clusters A-U with their respective MIRU profiles allowed the identification of 12 subclusters, grouping a total of 34 resistant strains as follows: subcluster A1 (*n* = 2; SIT53/MIT382), B1 (*n* = 32; SIT50/MIT42), D1 (*n* = 2; SIT42/MIT810), G1 (*n* = 6; SIT20/MIT307), G2 (*n* = 3; SIT20/MIT1048), H1 (*n* = 3; SIT93/MIT25), L1 (*n* = 2; SIT45/MIT23), L2 (*n* = 2; SIT45/MIT34), N1 (*n* = 5; SIT92/MIT3), O1 (*n* = 4; SIT129/MIT1089), and P1 (*n* = 2; SIT5/MIT15). MDR isolates (*n* = 6/19) were grouped in subclusters G2, H1, and P1, containing a total of *n* = 8 resistant isolates (G2 also contained an INH monoresistant strain and H1 included a RIF monoresistant strain). The clustered MDR isolates had been obtained in Guadeloupe (*n* = 2) and French Guiana (*n* = 4) between 1999 and 2006. The 2 MDR isolates of subcluster P1 also displayed resistance to streptomycin and ethambutol and the isolates of subcluster G2 belonged to SIT20; one of the 4 spoligotypes was found to be associated with drug resistance.

A total of 13 INH monoresistant isolates were grouped in 3 clusters (Table 4), A1 (*n* = 2, SIT53/MIT382), G1 (*n* = 6, SIT20/MIT307), and N1 (*n* = 5, SIT92/MIT3). Most of them (*n* = 12/13) were isolated in French Guiana between 1998 and 2009 and might reflect cases of active transmission in this department. Indeed, the spoligotype SIT92 harbored by the strains of subcluster N1 seems to be associated with INH monoresistance since 75% of the strains with this SIT profile were associated with this resistance pattern in our study. Subcluster A1 (*n* = 2) might further include some of the nine SIT53 INH monoresistant strains isolated from 1999 to 2003 in French Guyana (*n* = 8/9; 1 isolate from Guadeloupe), although unfortunately no MIRU profiles were available for these strains; the remaining isolates showed streptomycin resistance and might not be linked. Other clusters included SM-resistant subcluster L1 (*n* = 2, SIT45/MIT23, mean patient age 34.5) and L2 (*n* = 2, SIT45/MIT34, mean patient age 51 years) in Martinique and Guadeloupe, respectively, and subclusters B1 (*n* = 3, SIT50/MIT42, 2 patients from Guadeloupe and 1 from French Guiana) and O1 (*n* = 4, SIT129/SIT1089, all patients from French Guiana) with heterogeneous drug-resistance profiles (for a detailed description of all subclusters, readers are kindly referred to Table 4).

**Figure 2:**
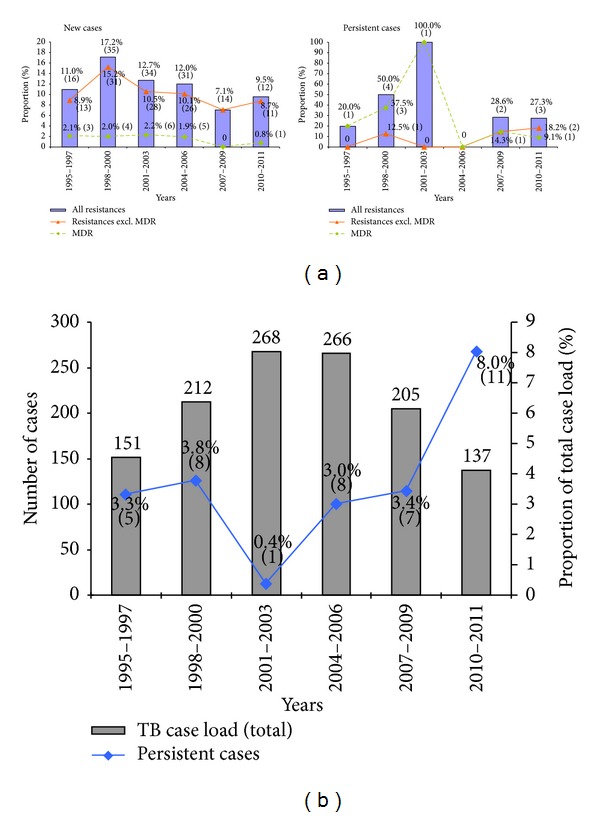
Trends in long-term distribution of drug resistance (total, non-MDR, and MDR) observed among new cases versus persistent cases (a) as well as in terms of total case load versus persistent cases (b). The data is grouped in three-year time periods (with the exception of the last 2-year period for 2010-2011) to facilitate observation of trends.

As far as the long-term development of drug resistance is concerned, the results obtained are illustrated in function of drug resistance (total, non-MDR, and MDR) observed among new cases versus persistent cases in Figure 2(a), as well as in terms of total case load versus persistent cases (Figure 2(b)). This data is grouped in 3-year periods (with the exception of the last 2-year period for 2010-2011) to facilitate observation of trends. As can be seen from this data, the proportion of drug-resistant and MDR strains among new cases steadily decreased from 1998–2000 to 2007–2009, followed by a slight increase of the ratio of drug-resistant isolates among new cases in 2010-2011. In contrast, the proportion of resistant and MDR strains amongst persistent cases did not show any tendencies. While the number of MDR strains is clearly inferior to that of strains presenting other forms of resistances among new cases (*n* = 19 versus 123, resp.), the opposite is true for persistent cases or relapses (*n* = 7 versus 4, resp.).

## 4. Discussion

Although underreporting of TB notification rate is well-known due to incompleteness of notification registers as well as relatively lower reporting of immigrant cases [14], the centralization of the TB bacteriological services for the three French Departments of the Americas allowed having a better global assessment of the TB situation in our setting. The data presented in this study showed that the majority of the TB cases in the FDA occurred in French Guiana (*n* = 716/1239, 57.8%, Table 1). Even though this observation is based on culture positive isolates only, it is consistent with incidence data for the 3 overseas French departments (between 10.2 and 44 cases/100,000 inhabitants during the study period) inferred from case notification rates showing a slow but gradual decrease in TB cases [15–17]. Nevertheless, a slight increase of TB incidence was observed between 2009 and 2011 in Guadeloupe and Martinique [18], which could be linked to the arrival of important numbers of refugees following the earthquake in Haiti in January 2010 [19].

We attempted applying international criteria to define TB relapses (American Thoracic Society, 1999); nonetheless, we had to resort to an approximate determination of “persistent” cases as described in the methods section due to the unavailability of certain clinical data, rendering it slightly difficult to interpret our results in context to the situation prevailing in metropolitan France. Nevertheless, regarding drug resistance, persistent cases in our study show characteristics similar to the relapse cases on the national level. For the period of 2010-2011, the proportion of resistant and MDR cases observed in France was 10.7% and 2.1%, respectively, for all cases and 18.4% and 8.5% for relapses [20, 21], which is comparable to the rates observed in our setting during the same period (10.9% and 1.5% for all cases versus 27.3% and 9.1% for persistent cases). Likewise, the proportion of persistent cases in the French Departments of the Americas (8.0% for 2010-2011; 3.2% for the whole study period) is in accordance with the relapse quota of 8.3% estimated for France for 2010-2011 [21]. With relapses being a major risk factor for the development of secondary multi-drug resistance [22], it is essential to thoroughly identify and further characterize the subpopulation of patients suffering from a TB relapse in the 3 French Departments of the Americas in future studies.

Regarding the *M. tuberculosis* drug resistance profiles, we observed that resistance to at least INH or SM occurred most frequently (7.0% and 5.5%, resp., among new cases), which is in agreement with data reported from numerous other countries including France (primary SM resistance in 2004–2011: 6.1% [21, 23, 24]) and China [25]. Comparing primary SM resistance in the three departments, Martinique had a significantly higher rate than Guadeloupe and French Guiana (9.6% versus 4.0% and 5.0%, resp., *P* = 0.03). In combination with the high mean age of patients from Martinique, this supports the hypothesis that most TB cases in Martinique are due to the reactivation of old infections [26]. As SM is no longer used as a first-line anti-TB drug in France [27], detecting resistance to this particular antibiotic allows for estimating the proportion of TB reactivation on the one hand—if the patients are old—and for assessing the rate of active transmission of these strains on the other hand—if the patients are young. Lastly, the low number of isolates displaying resistance to 3 or 4 first-line drugs and the total absence of these resistance profiles among persistent cases are both very encouraging. To date, no mutation conferring resistance to several antibiotics at a time has been identified in *M. tuberculosis* [28]. Hence, multiple drug resistance is considered the result of an accumulation of a number of mutations which in turn is only possible if the resistant strain is inadequately treated and actively transmitted. Therefore, the low rate of cumulated resistances in the study testifies to limited transmission of drug-resistant *M. tuberculosis* strains in the French departments indicating appropriate medical care. Considering the fact that 65.4% (*n* = 17/26) of the MDR cases were smear-positive (and therefore possibly contagious), this is an encouraging observation.

Regarding genotypic resistance, the most commonly observed MTBDR*plus* hybridization pattern was the absence of hybridization of the WT2 probe (*n* = 3/5 strains). This is noteworthy as mutations of these codons are rare compared to other mutation hotspots like codons 526 and 531 [29–32]. Even more astounding is the absence of the WT4 hybridization signal combined with the presence of both WT3 and WT5, observed in 2/5 strains, as all codons that are covered by WT4 are also covered by either WT3 or WT5. The exact nature of the *rpoB* mutations in all four strains not hybridizing with the WT2 and/or WT4 probe should be ideally clarified by *rpoB* sequencing in near future.

Regarding TB/HIV coinfection (HIV serology results available for 512/1239 or 41.3% of patients), our study showed an alarmingly high global rate of 47.5% coinfection in the three departments; however, the rates varied from high of 55.9% (*n* = 180/322) in French Guiana and 48.5% (*n* = 180/322) in Guadeloupe to a lower rate of 14.9% (*n* = 13/87) in Martinique; *P* < 0.001—which is in accordance with the rates of positive HIV serology/1000 tests performed in the three departments (8.7, 5.0, and 2.0/1000 for French Guiana, Guadeloupe, and Martinique, resp., [33]). It was reported that the proportion of migrants and people with precarious living condition was higher in Guadeloupe among HIV positive patients than that observed in Martinique [34]. As AIDS considerably influences the epidemiology of TB, these findings should be kept in mind where treatment and follow-up of TB patients is concerned. Nonetheless, positive HIV serology did not show any correlation to the drug susceptibility or resistance profile of the isolates in our study (Table 1).

Lastly, regarding circulating genotypes and *M. tuberculosis* drug resistance profiles in a given study area, recent studies have suggested the importance of the genetic background of the MTBC clones in circulation in that particular zone [35, 36]. In our study, two lineages, X and LAM, were overrepresented in drug-resistant and MDR-TB cases, respectively. Indeed, the clustering rate of isolates belonging to the LAM and X lineages was higher than for other phylogenetic families (91.2% versus 85.6%, *P* < 0.025, and 93.8% versus 86.4%, *P* = 0.06 of clustered isolates, resp.), suggesting a more important active transmission of these two lineages which could indirectly favor the apparition and spread of resistances. In contrast, the Beijing clade, having been linked with drug resistance in numerous studies from all over the world [35, 37, 38], was not found connected to increased drug resistance in study area, even though it has been isolated regularly since 1995 [26, 39].

Out of a total of 19 predominant spoligotypes identified for the 1239 isolates of *M. tuberculosis* of the study, four patterns corresponding to SIT20/LAM1, SIT64/LAM6, SIT45/H1, and SIT46/undefined lineage were significantly associated with drug resistance. Studying the transmission chains of these 4 spoligotypes showed a strong association with drug resistance (Table 4). An active transmission of SIT 20 in French Guiana since 1999 could be established since 2 subclusters identified based on the VNTR profiles (G1 and G2) differed by a single locus, locus 26 (12MIRU profile G1: 223226163321; G2: 223226193321). It is therefore probable that these single locus variants (SLV) belong to the same transmission chain of INH monoresistant (subcluster G1) and MDR (subcluster G2) strains. Indeed, it was shown that epidemiological links between patients infected by strains differing by a SLV are more likely than for patients whose strains differ in more than one locus [40, 41]. Consequently, patients infected by strains of SIT20/LAM1 pattern should be subject to specific surveillance in our setting. Alarmingly, this spoligotype is encountered in numerous other countries of our region: Venezuela, 2.7% [42]; Brazil, 6.1% [43]; Colombia, 1.5%; Haiti, 5.9%; and Jamaica, 3.3% ([7] and SITVIT2 database), and such an active circulation of this clone in the area might favor the emergence of resistances. According to the SITVIT2 database, SIT20/LAM1 is phylogeographically linked to Portugal in Europe, where it accounts for 11.7% of all spoligotypes found.

Regarding the other resistance associated spoligotype patterns in our study, the TB case characteristics indicate reactivation with low transmission for SIT45/H1 (2 subclusters with 2 patients each) or isolated cases of short-lived transmission for SIT46 of undefined lineage (8 patients, all diagnosed in 1999). The presence of SIT45 which is known to be implicated in 30% of the TB cases in the neighboring island of St. Lucia (according to SITVIT2), both in Guadeloupe and Martinique but not French Guiana, illustrates existing links between the three islands. Since four spoligotype profiles were found to harbor higher proportions of drug resistant strains in the FDA (SIT20, SIT45, SIT46, and SIT64), we also interrogated the SITVIT2 database to see if these patterns were associated with drug-resistance in other settings. SIT20 and SIT45 showed similar or higher proportions of drug resistance among isolates of worldwide origin as compared to rates observed in the FDA (SIT20: 48.3% of drug-resistant isolates in the present study versus 46.7% in SITVIT2 (*n* = 84/180); SIT45: 31.3% versus 55.6% in SITVIT (*n* = 20/36)), while the 2 remaining SITs (i.e., SIT46 and SIT64) showed lower proportion of resistance elsewhere than in the FDA.

Finally, if combination of spoligotypes with 12-loci MIRU-VNTR profiles has permitted the identification of 12 subclusters including 34 drug-resistant *M. tuberculosis* isolates, the available epidemiological data was far too scarce to precisely estimate their level of active transmission among respective population of the FDA. This limitation is also observed concerning importation and transmission of *M. tuberculosis * clones phylogeographically linked to surrounding countries of the region (i.e., Guyana, Suriname, Haiti, and St. Lucia). Nonetheless, it was shown that migratory flows from the neighboring high incidence countries Suriname and Brazil lead to the import (and circulation) of *M. tuberculosis* strains phylogeographically associated with these countries in French Guiana [26].

The most notable examples are SIT131/T1 and SIT1340/EAI6-BGD1, both linked to Suriname (and its western neighbor Guyana) and isolated almost exclusively in French Guiana in the present study (Guianese isolates: SIT131, *n* = 44/47; and SIT1340, *n* = 16/16). As these SITs are implicated in drug resistance (9.8% and 7.1% resistant isolates in Suriname, resp., according to SITVIT2 database), their importation into French Guiana is likely to entrain an introduction of resistant strains in the department. While this hypothesis holds true for SIT1340 (resistant isolates, *n* = 2/18 or 11.1%), none of the strains with SIT131 displayed any resistance. Considering the fact that the Caribbean comprises a number of countries with a high or extremely high TB incidence with a significant proportion of MDR-TB [44–48], for example, Haiti (incidence: 230/100,000; MDR: 2.9% of new cases), the Dominican Republic (incidence: 67/100,000; MDR: 6.6% of new cases), Guyana (incidence: 111/100,000; MDR: 12% of new cases), Suriname (incidence: 145/100,000; MDR unreported), or Brazil (incidence: 43/100,000; MDR: 4.2% of new cases), and whose populations constantly interact with the inhabitants of the three French Departments of the Americas, the results of our study on the emergence and transmission of drug-resistant and MDR clones (genotypes) of *M. tuberculosis* are very encouraging.

## 5. Conclusion

Although the proportion of drug-resistant and MDR *M. tuberculosis * isolates is stable at a moderate level in the French Departments of the Americas, a continued surveillance of drug resistance accompanied by universal genotyping of the isolates should be maintained for the following reasons: (i) import of *M. tuberculosis* including drug-resistant and MDR strains from high incidence neighboring countries; (ii) possible resurgence of increased drug-resistance rates following the increase of persistent cases in 2010-2011; and (iii) the presence and ongoing transmission of four genotypes associated with drug resistance (SIT20/LAM1, SIT45/H1, SIT46/undefined lineage, and SIT64/LAM6). To accomplish this task and to strengthen local anti-TB programs, the regional surveillance network should further include 2 remaining French territories of the region: St. Martin and St. Barthelemy.

## Supplementary Material

Detailed listing of the spoligotype patterns obtained for the 1239 Mycobacterium tuberculosis complex (MTBC) strains, split into 2 separate tables showing patterns obtained for drug susceptible (*n* = 1086; Supplemental Table S1) and drug-resistant isolates (*n* = 153; Supplemental Table S1). Each pattern is listed by its frequency of isolation; the tables also provide the corresponding lineage, SIT number, and proportion (%) in the study. Note that percentages were calculated relative to the total number of drug susceptible isolates in Table S1 and relative to the total number of drug resistant isolates in Table S2. Orphan profiles indicate patterns that were unique in the SITVIT2 database.

## Figures and Tables

**Table 1:** Characteristics of the 1239 patients and the corresponding *M. tuberculosis* isolates; data on cases of resistant and multiresistant TB are specified in the respective columns.

Variables	Resistant TB (*n* = 153; 12.3%)		MDR-TB (*n* = 26; 2.1%)		*N*_ total_^ a^ (%) (*n* = 1239)
	*N* (%)	*P*	*N* (%)	*P*	
Gender					
Male	101 (66.5)	*0.651 *	13 (50.0)	*0.147 *	786 (64.4)
Female	51 (33.5)		13 (50.0)		434 (35.6)
*Unknown* ^b^	*1 *		*0 *		*19 *
Age					
<15 years	7 (4.8)	*0.331 *	0	*0.912 *	46 (3.9)
15–65 years	126 (85.7)		23 (88.5)		982 (82.9)
>65 years	14 (9.5)		3 (11.5)		156 (13.2)
*Unknown* ^b^	*6 *		*0 *		*55 *
Mean age	41.2	*0.724 *	40.9	*0.715 *	42.6
Origin^c^					
Guadeloupe	39 (25.5)	*0.755 *	8 (30.8)	*0.331 *	346 (27.9)
Martinique	23 (15.0)		1 (3.9)		177 (14.3)
French Guiana	91 (59.5)		17 (65.4)		716 (57.8)
HIV status					
Positive	33 (51.6)	*0.506 *	13 (65.0)	*0.117 *	243 (47.5)
Negative	31 (48.4)		7 (35.0)		269 (52.5)
*Unknown* ^b^	*89 *		*6 *		*727 *
Bacteriology^d^					
Smear +/culture+	77 (53.5)	*0.530 *	17 (65.4)	*0.425 *	646 (56.2)
Smear −/culture+	67 (46.5)		9 (34.6)		503 (43.8)
*Unknown* ^b^	*9 *		*0 *		*90 *
Case characteristic					
Persistent	11 (7.2)	*0.007 *	7 (26.9)	*<0.001 *	40 (3.2)
New case	142 (92.8)		19 (73.1)		1199 (96.8)
Lineage^d^					
LAM	38 (24.8)	*0.060 *	11 (42.3)	*0.470 *	294 (23.7)
H	27 (17.7)		5 (19.2)		275 (22.2)
T	39 (25.5)		7 (26.9)		373 (30.1)
EAI	10 (6.5)		1 (3.9)		89 (7.2)
X	16 (10.5)		1 (3.9)		81 (6.5)
Others	23 (15.0)		1 (3.9)		127 (10.3)

^a^N_total_: total number of patients per variable.

^b^Number of patients/isolates for which this information was not available (excluded from calculations).

^c^Department where a given *M. tuberculosis *isolate has been obtained.

^d^LAM: Latin-American-Mediterranean; H: Haarlem; EAI: East-African-Indian; others: lineages/MTBC members accounting for <1.5% of the isolates (*M. africanum*, Beijing, *M. bovis*, CAS: Central-Asian, LAM10-CAM, LAM7-TUR, S) and phylogenetically undefined spoligotype profiles.

**Table 2:** Observed resistance profiles as a function of the case characteristics (new versus persistent).

Resistance profiles	New case *N* (%)	Persistent *N* (%)	*P*	Total *N* (%)
	*N*_*T*_ = 1199	*N*_*T*_ = 40		
Resistant to INH*	84 (7,0)	9 (22,5)	*0,002 *	93 (7,5)
Resistant to RIF*	27 (2,3)	8 (20,0)	*<0,001 *	35 (2,8)
Resistant to EMB*	6 (0,5)	0	*1,000 *	6 (0,5)
Resistant to SM*	66 (5,5)	1 (2,5)	*0,720 *	67 (5,4)
Resistant to PZA*	13 (1,1)	0	*1,000 *	13 (1,1)

Monoresistant to INH**	50 (4,2)	2 (5,0)	*0,683 *	52 (4,2)
Monoresistant to RIF**	7 (0,6)	1 (2,5)	*0,231 *	8 (0,7)
Monoresistant to EMB**	0	0		0
Monoresistant to SM**	40 (3,3)	1 (2,5)	*1,000 *	41 (3,3)
Monoresistant to PZA**	4 (0,3)	0	*1,000 *	4 (0,3)

Resistant to 1 AB***	101 (8,4)	4 (10)	*0,770 *	105 (8,5)
Resistant to 2 AB***	32 (2,7)	7 (17,5)	*<0,001 *	39 (3,1)
Resistant to 3 AB***	5 (0,4)	0	*1,000 *	5 (0,4)
Resistant to 4 AB***	4 (0,3)	0	*1,000 *	4 (0,3)

*comprises all isolates resistant to the respective drugs (irrespective of other associated resistances).

**Isolates resistant to only INH (isoniazid), RIF (rifampicin), EMB (ethambutol), SM (streptomycin), or PZA (pyrazinamide), respectively.

***Strains resistant to 1, 2, 3, or 4 of the tested antibiotics (AB), no strain displayed resistance to all drugs.

**Table 3:** MTBDR*plus rpoB* probe-hybridization patterns for of the 5 tested MDR strains.

Nonhybridized WT probes	Hybridization with mutation probes
WT 2; WT 4; WT 6–8 not tested*	—
WT 4; WT 6–8 not tested	MUT 2B
WT 2	—
WT 8	MUT 3
WT 2	—

*Not tested: the probes of WT 6–8 were not included in this version of MTBDR*plus* kit (note that this ancient version of the MTBDR*plus* kit did not include probes for *inhA* mutations).

**Table 4:** Clusters (*n* = 21) of new cases containing at least 2 drug-resistant strains and corresponding drug-resistance profiles.

Cluster designation^a^	SIT designation	Number of isolates^b^	Number and proportion (%) of drug-resistant isolates^c^	Drug-resistance profile^d^	Year	Origin^d^	MIT designation	VNTR defined subclusters^e^
A	53	132	20 (15.2%)	S	1997	GLP		*(A2) *
				S	1999	GUF	8	
				S	2004	GUF	33	
				S	2004	GLP	802	
				S	2011	GLP	1131	
				I	1999	GLP	13	
				I	1999	GUF		*(A3) *
				I	1999	GUF		
				I	2000	GUF		
				I	2001	GUF		
				I	2001	GUF		
				I	2002	MTQ		
				I	2002	MTQ		
				I	2003	GUF		
				I	2004	GUF	382	**A1**
				I	2008	GUF	382	
				I	2008	GUF	1459	
				S, I	2002	GUF		*(A4) *
				S, I	1996	GLP		
				S, I, R	2005	MTQ	32	

B	50	110	5 (4.5%)	S	2003	GUF		
				S	2004	GUF	42	B1
				I	2003	GLP	42	
				R	2008	GLP	42	
				S, I	2003	GLP	184	

C	42	60	2 (3.3%)	S, I, R, E	2003	GUF		
				I	2005	GLP	25	

D	2	42	7 (16.7%)	I	1996	GUF		*(D2) *
				I	2010	GLP	350	
				I, R	1996	GUF		*(D3) *
				I, R	1997	GUF		
				S, I	1998	MTQ		
				S, I	2005	GUF	810	**D1**
				S, I	2006	GUF	810	

E	17	34	2 (5.9%)	I, R, E	1999	GLP	1542	
				R	2011	GUF	26	

F	14	31	6 (19.4%)	S	1997	GLP		*(F1) *
				S	1997	GLP		
				S	1997	GLP		
				S	2010	GLP	24	
				I	1998	GLP	28	*(F2) *
				I	2000	MTQ		

G	20	29	14 (48.3%)	S	2009	GUF	25	
				I	2001	GUF		
				I	2001	GUF		
				I	2004	GLP	307	**G1**
				I	2004	GUF	307	
				I	2004	GUF	307	
				I	2006	GUF	307	
				I	2006	GUF	307	
				I	2009	GUF	307	
				I	1999	GLP	1048	**G2**
				S, I, R	2000	GUF	1048	
				S, I, R	2001	GUF	1048	
				I, R	2001	GUF		*(G3) *
				I, R	2002	GUF		

H	93	27	4 (14.8%)	S, I	1997	GUF		
				I, R	1999	GLP	25	**H1**
				I, R	2006	GUF	25	
				R	2004	GLP	25	

I	64	24	6 (25.0%)	S, P	1997	GUF		*(I1) *
				S, P	1999	GUF		
				S, P	2001	GUF		
				S, P	2001	GUF		
				S, P	2002	GUF		
				S	2006	GUF	328	

J	1	17	3 (17.6%)	S, I	2002	GUF		*(J1) *
				S, I	2002	GUF		
				S, I	2003	GUF		

K	46	16	8 (50.0%)	S	1999	MTQ		*(K1) *
				S	1999	MTQ		
				S	1999	MTQ		
				S	1999	MTQ		
				S	1999	MTQ		
				S	1999	MTQ		
				S	1999	MTQ		
				S	1999	MTQ		

L	45	16	5 (31.3%)	S	2004	MTQ	23	**L1**
				S	2009	MTQ	23	
				S	2008	GLP	34	**L2**
				S	2011	GLP	34	
				S	2008	MTQ	Orphan	

M	1340	15	2 (13.3%)	I, R	2000	GUF		
				R	2004	GUF	798	

N	92	8	6 (75.0%)	I	1998	GUF	3	**N1**
				I	1998	GUF	3	
				I	1998	GUF	3	
				I	2005	GUF	3	
				I	2009	GUF	3	
				I	2001	GUF		

O	129	8	4 (50.0%)	P	1996	GUF	1089	**O1**
				S	2008	GUF	1089	
				S	2011	GUF	1089	
				S	2011	GUF	1089	

P	5	8	3 (37.5%)	S, I, R, E	2005	GLP	15	**P1**
				S, I, R, E	2006	GUF	15	
				I, E	2008	GUF	Orphan	

Q	334	5	2 (40.0%)	S, I, R	2002	GUF		
				I	2003	GUF		

R	482	4	4 (100%)	P	1996	GLP		*(R1) *
				P	1998	GLP		
				P	2004	GUF	49	
				I, P	2010	GUF	281	

S	75	4	2 (50.0%)	I	2003	GLP		
				I, R	2004	GUF	42	

T	385	3	2 (50.0%)	I	1999	GUF		*(T1) *
				I	2003	MTQ		

U	2884	2	2 (100%)	S	1999	MTQ		*(U1) *
				S	2006	MTQ	25	

^a^ *n* = 21/115 clusters (labeled A-U) identified based on spoligotyping results of new cases included at least 2 resistant isolates.

^b^In total, 595 isolates are grouped in the 21 clusters defined in^a^.

^c^ *n* = 109/595 clustered cases are caused by resistant strains.

^d^I: isoniazid, R: rifampicin, S: streptomycin, E: ethambutol, P: pyrazinamide ^d^GLP: Guadeloupe, MTQ: Martinique, GUF: French Guiana.

^e^Subclusters defined by spoligotypes and MIRU-VNTR profiles (SIT and MIT); subclusters in parentheses were defined based on resistance profiles only (incomplete or missing MIRU data), while those in bold were defined based on both molecular typing and drug-resistance profiles.
