# Supplementary material for: A Systematic Follow-Up of Mycobacterium tuberculosis Drug-Resistance and Associated Genotypic Lineages in the French Departments of the Americas over a Seventeen-Year Period
Source: Biomed Res Int. 2014 Mar 13;2014:689852. doi: 10.1155/2014/689852 (PMC3971487; doi:10.1155/2014/689852)
Supplement: Supplementary file 1 — Detailed listing of the spoligotype patterns obtained for the 1239 Mycobacterium tuberculosis complex (MTBC) strains, split into 2 separate tables showing patterns obtained for drug susceptible (n = 1086; Supplemental Table S1) and drug-resistant isolates (n = 153; Supplemental Table S1). Each pattern is listed by its frequency of isolation; the tables also provide the corresponding lineage, SIT number, and proportion (%) in the study. Note that percentages were calculated relative to the total number of drug susceptible isolates in Table S1 and relative to the total number of drug resistant isolates in Table S2. Orphan profiles indicate patterns that were unique in the SITVIT2 database. [file 689852.f1.pdf]

SUPPLEMENTAL TABLE S1: Spoligotypes obtained for drug susceptible *M. tuberculosis* isolates (n=1086).

Lineage	SIT	Spoligotype	Nb (%)*
T1	53	■■■■■■■■■■■■■■■■■■■■■■■■■■■■■■■■□□□□■■■■■■■	114 (10.5)
H3	50	■■■■■■■■■■■■■■■■■■■■■■■■■■■■■■□■□□□□■■■■■■■	109 (10.04)
LAM9	42	■■■■■■■■■■■■■■■■■■■■□□□□■■■■■■■■□□□□■■■■■■■	59 (5.43)
T1	131	■■■■■■■■■■■■□□■■■■■■■■■■■■■■■■■■□□□□■■■■■■■	47 (4.33)
H2	2	□□□□□□□□□□□□□□□□□□□□□□□□■□□□□□□■□□□□■■■■■■■	36 (3.31)
LAM2	17	■■□■■■■■■■■■□■■■■■■■□□□□■■■■■■■■□□□□■■■■■■■	32 (2.95)
X3	14	■□■□□□□□□□□□■■■■■□■■■■■■■■■■■■■■□□□□■■■■■■■	25 (2.3)
LAM5	93	■■■■■■■■■■■■□■■■■■■■□□□□■■■■■■■■□□□□■■■■■■■	24 (2.21)
T1	51	■■■■■■■■■■■■■■■■■■■■■■■■■■■■■■■■□□□□■■■□□□□	21 (1.93)
LAM6	64	■■■■■■■■■■■■■■■■■■■■□□□□■■■■□■■■□□□□■■■■■■■	18 (1.66)
LAM1	20	■■□■■■■■■■■■■■■■■■■■□□□□■■■■■■■■□□□□■■■■■■■	15 (1.38)
Beijing	1	□□□□□□□□□□□□□□□□□□□□□□□□□□□□□□□□□□■■■■■■■■■	14 (1.29)
H1	47	■■■■■■■■■■■■■■■■■■■■■■■■■□□□□□□■□□□□■■■■■■■	14 (1.29)
H1	62	■■■■■■■■■■■■■■■■■■■■■■■■■□□□□□□■□□□□■■■□■■■	14 (1.29)
X3	91	■■■□□□□□□□□□□■■■■□■■■■■■■■■■■■■■□□□□■■■■■■■	14 (1.29)
EAI6-BGD1	1340	■■■■■■■■■■■■■■■■□■■■■■□■■■■■□□□□■□■■□■■■■■■	14 (1.29)
H1	45	■■■■■■■■■■■■■■■■■■■■■■■□■□□□□□□■□□□□■■■■■■■	12 (1.1)
EAI1-SOM	72	■■■■■■■■■■□■■■■■■■■■■■■■■■■□□□□□□□■■■■■□■■■	12 (1.1)
T5	44	■■■■■■■■■■■■■■■■■■■■■■□■■■■■■■■■□□□□■■■■■■■	10 (0.92)
Undefined	4	□□□□□□□□□□□□□□□□□□□□□□□□■■■■■■■■□□□□■■■■■■■	8 (0.74)
T1	7	□■■■■■■■■■■■■■■■■■■■■■■■■■■■■■■■□□□□■■■■■■■	8 (0.74)
S	34	■■■■■■■■□□■■■■■■■■■■■■■■■■■■■■■■□□□□■■■■■■■	8 (0.74)
Undefined	46	■■■■■■■■■■■■■■■■■■■■■■■■□□□□□□□□□□□□□□□□□□□	8 (0.74)
H3	67	■■■■■■■■■■■■■■■■■■□□□□■■■■■■■■□■□□□□■■■■■■■	8 (0.74)
T	73	■■■■■■■■■■■■□■■■■■■■■■■■■■■■■■■■□□□□■■■□■■■	8 (0.74)
T1	77	■■■■□□□■■■■■■■■■■■■■■■■■■■■■■■■■□□□□■■■■■■■	8 (0.74)
X3	70	■■■□□□□□□□□□■■■■■□■■■■■■■■■■■■■■□□□□■■□■■■■	7 (0.64)
LAM5	1337	■■■■■■■■■■■■□□□□□□■■□□□□■■■■■■■■□□□□■■■■■■■	7 (0.64)
T1	63	□■■■■■■■■■■■■■■■■■■■■□■■■■■■■■■■□□□□■■■■■■■	6 (0.55)
T4-CEU1	94	■■■■■■■■■■■■■■■■■■□■■■□□■■■■□■■■□□□□■□□■■■■	6 (0.55)
EAI7-BGD2	96	■■■■■■■■■■■■■■■■■■■■■■□■□□□□□□□□□□■■□■■■■■■	6 (0.55)
LAM2	193	■■□■■■■■■■■■□■■■■■■■□□□□■■■■■□■■□□□□■■■■■■■	6 (0.55)
Undefined	2508	■■■■■■■■■■□■■■■■■■■■□□□□□□□□□□□□□□□□□□□□□□□	6 (0.55)
T1	5	□□□□□□□□□■■□■■■■■■■■■■■■■■■■■■■■□□□□■■■■■■■	5 (0.46)
LAM	29	■■■■■□□□□□□□□□□□□■■□□□□□□□□□□□□□□□□□□■■■■■	5 (0.46)
LAM3	33	■■■■■■■■□□□■■■■■■■■■□□□□■■■■■■■■□□□□■■■■■■■	5 (0.46)
T4	40	■■■■■■■■■■■■■■■■■■□■■■■■■■■■■■■■□□□□■■■■■■■	5 (0.46)
X1	119	■■■■■■■■■■■■■■■■■□■■■■■■■■■■■■■■□□□□■■■■■■■	5 (0.46)
LAM	133	■■■■■■■■■■■□□□□□■■■□□□□□■□■■■■■■□□□□■□□□■■□	5 (0.46)
X2	137	■■■■■■■■■■■■■■■■■□■■■■■■■■■■■■■■□□□□■■□□□□■	5 (0.46)
LAM6	176	■■■■■■■■■■■■□■■■■■■■□□□□■■■■□■■■□□□□■■■■■■■	5 (0.46)
Undefined	1084	■■□□□□□□□□□□□□□□□□□□□□□□□□□□■■■■□□□□■■■■■■■	5 (0.46)
H3	3	□□□□□□□□□□□□□□□□□□□□□□□□■■■■■■□■□□□□■■■■■■■	4 (0.37)
EAI3-IND	11	■□□■■■■■■■■■■■■■■■■■■■■■■■■■□□□□■□■■□□□■■■■	4 (0.37)
EAI2-Manila	19	■■□■■■■■■■■■■■■■■■■□□■■■■■■■□□□□■□■■■■■■■■■	4 (0.37)
T	31	■■■■■■■□□□□□□■■■■■■■■■■■■■■■■■■■□□□□■■■□□□□	4 (0.37)
H3	49	■■■■■■■■■■■■■■■■■■■■■■■■■■■■■■□■□□□□■■■□■■■	4 (0.37)
T2	52	■■■■■■■■■■■■■■■■■■■■■■■■■■■■■■■■□□□□■■■□■■■	4 (0.37)
LAM4	60	■■■■■■■■■■■■■■■■■■■■□□□□■■■■■■■■□□□□■■■□■■■	4 (0.37)
LAM6	95	■■■■■■■■■■■■■■■■■■■■□□□□■■■■□■■■□□□□■■■□■■■	4 (0.37)
H3	99	■■■■□■■■■■■■■■■■■■■■■■■■■■■■■■□■□□□□■■■■■■■	4 (0.37)
EAI6-BGD1	129	■■■□□□□□□■■■■■■■■■■■■■□□■■■■□□□□■□■■■■■■■■■	4 (0.37)
LAM5	136	■■■■■■■■■■■□□□□□■■■□□□□□■□■■■■■■□□□□■□□■■■■	4 (0.37)
T4	259	■■■■■■■□■■■■■■■■■■□■■■■■■■■■■■■■□□□□■■■■■■■	4 (0.37)
LAM8	290	■■■■■■■■■■■■■■■■■■■■□□□□■■□■■■■■□□□□■■■■■■■	4 (0.37)
H3	390	■■■■■■■■■■■■■■■■■■■■■■■■■■■■■□□■□□□□■■■■■■■	4 (0.37)
EAI5	924	■■■■■■■■■■■□□□□□□□□□□□□□■■■■□□□□■□■■□■■■■■■	4 (0.37)
T1	2791	■■■■■■■■■■■■■■■■■■■■■■■■□□□■■■■■□□□□■■■■■■■	4 (0.37)
Undefined	32	■■■■■■■■□□□□□□□□□□□□□□□□□□□□□□□□□□□□□□■■■■■	3 (0.28)
T3	37	■■■■■■■■■■■■□■■■■■■■■■■■■■■■■■■■□□□□■■■■■■■	3 (0.28)
Undefined	56	■■■■■■■■■■■■□■■■■■■■■■■■□□□□□□□□□□□□□□□□□□□	3 (0.28)
LAM11-ZWE	59	■■■■■■■■■■■■■■■■■■■■□□□□■■□□□□■■□□□□■■■■■■■	3 (0.28)
S	88	■■■■■■■■□□■■□■■■■■■■■■■■■■■■■■■■□□□□■■■□■■■	3 (0.28)
H1	103	■■■■■■■■■■■■■■■■■■■■■■□■■□□□□□□■□□□□■■■□■■■	3 (0.28)
LAM9	177	□■■■■■■■■■■■■■■■■■■■□□□□■■■■■■■■□□□□■■■■■■■	3 (0.28)
T1	334	■□■■■■■■■■■■■■■■■■■■■■■■■■■■■■■■□□□□■■■■■■■	3 (0.28)
EAI1-SOM	349	■■■■■■■■■■■■□■■■■■■■■■■■■■■■□□□□■□■■■■■□■■■	3 (0.28)
LAM1	578	■■□□■■■■■■■■■■■■■■■■□□□□■■■■■■■■□□□□■■■■■■■	3 (0.28)
LAM6	1066	■■■■■■■■■■■■■■□■■■■■□□□□■■■■□■■■□□□□■■■■■■■	3 (0.28)
T1	1526	■■■□□□□□□□□□□■■■■■□□■■■■■■■■■■■■□□□□■■■■■■■	3 (0.28)
T1	2437	■■■■■■■■■□□□■■■■■■■■■■■■■■■■■■■■□□□□■■■■■■■	3 (0.28)
LAM6	2521	■■■□■■■■■■■■■■■■■■■■□□□□■■■■□■■■□□□□■■■■■■■	3 (0.28)
H3	2980	■■■■■■■■■■■■■■□■■■■■■■■■■■■■□■□■□□□□■■■■■■■	3 (0.28)

LAM	3504	■■■■■■■■■■■□□■■■■■■■□□□□■■■■□■■■□□□□■■■■■■■	3 (0.28)
Undefined	3506	■■■■■■■■■■■■□■■■■■■■□□□□■■■□□□□□□□□□■■■■■■■	3 (0.28)
EAI1-SOM	48	■■■■■■■■■■■■■■■■■■■■■■■■■■■■□□□□■□■■■■■□■■■	2 (0.18)
LAM10-CAM	61	■■■■■■■■■■■■■■■■■■■■■■□□□■■■■■■■□□□□■■■■■■■	2 (0.18)
T1	65	■■■■■■■■■■■■■■■■■■■■■■■■■■■■■■■■□□□□■□□■■■■	2 (0.18)
H3	66	■■■■■■■■■■■■■■■■■■□□□□■■■■■■■■□■□□□□■■□■■■■	2 (0.18)
H3	75	■■■■■■■■■■■■■■□■■■■■■■■■■■■■■■□■□□□□■■■■■■■	2 (0.18)
EAI8-MDG	76	■□□■■■■□□□□□□□□□□■□■■■■■■■■■□□□□■□■■■■■■■■■	2 (0.18)
T	78	■■■■■■■■■■■■■■■■■■■■■■■■■■■■■■■■□□□□■■■□□■■	2 (0.18)
X3	92	■■■□□□□□□□□□■■■■■□■■■■■■■■■■■■■■□□□□■■■■■■■	2 (0.18)
AFRI_2	101	■■■■■■■□□□□□■■■■■■■■□□□□■■□■■■■■□■■■□□□■■■■	2 (0.18)
T	102	■■■■■■■■■■■■□□□□■■■■■■■■■■■■■■■■□□□□■■■■■■■	2 (0.18)
T1	118	■■■■■■■■■■■■■■□■■■■■■■■■■■■■■■■■□□□□■■■■■■■	2 (0.18)
T1	122	■■■■■■■■■■■■■■■■■■■■■■■■■□■■■■■■□□□□■■■■■■■	2 (0.18)
Undefined	124	■■■■■■■■■■■■■■■■■■■■■■■■■■■■■■□□□□□□■■■■■■■	2 (0.18)
EAI5	126	■□□■■■■■■■■■■■■■■■■■■■■■■■■■□□□□■□■■■■■■■■■	2 (0.18)
LAM9	150	■■■■■■■■■■■■■■□■■■■■□□□□■■■■■■■■□□□□■■■■■■■	2 (0.18)
H3	168	■■■■■■■■■■■■■■■■■■■■■■■■■■■■■■□■□□□□■■□■■■■	2 (0.18)
AFRI_1	181	■■■■■■□□□■■■■■■■■■■■■■■■■■■■■■■■■■■■□■■■■	2 (0.18)
LAM1	195	■■□■■■■■■■■■■■■■■■■■□□□□■■■■■□■■□□□□■■■■■■■	2 (0.18)
T1	276	■■■■■■■■■■■■■■■■■■■■■■■■□□□□□□■■□□□□■■■■■■■	2 (0.18)
AFRI_1	326	■■■■■■□□□■■■■■■■■■■■■□□□■■■■■■■■■■■■■■□■■■■	2 (0.18)
LAM9	335	■■■■■■■■■■■■■■■■■■■■□□□□■■■■■□■■□□□□■■■■■■■	2 (0.18)
T1	393	■■■■■■■■■■■■■□■■■■■■■■■■■■■■■■■■□□□□■■■■■■■	2 (0.18)
T1	535	■■■■■■■■■■■■■■■■■■■■■□□□■■■■■■■■□□□□■■■■■■■	2 (0.18)
EAI1-SOM	958	■■■■■■■■■■■■■■■■■■■■■■■■■■□□□□□□■□■■■■■□■■■	2 (0.18)
H3	963	■■■■■■■■■■■■■■■■■■■■■□■■■■■■■■□■□□□□■■■■■■■	2 (0.18)
LAM	1086	■■■■□□□□□■■■□■■■■■■■□□□□■■■■■■■■□□□□■■■□■■■	2 (0.18)
LAM	1087	□□■■■■■■■■■■■■■■■■■■□□□□□■■■■■■■□□□□■■■■■■■	2 (0.18)
EAI3-IND	1762	■□□■■■■■■■■■■■■■■■■■□□■■■■■■□□□□■□■■□□□■■■■	2 (0.18)
LAM5	1999	■■■■■■■■■■■■□□□□□■■■□□□□■■■■■■■■□□□□■■■■■■■	2 (0.18)
S	2053	■■■■■■■■□□■■■■■■■■■■■■■■■□■■■■■■□□□□■■■■■■■	2 (0.18)
LAM	2310	■■■■■■■■□□□■■■■■■■■■□□□□■■□■■■■■□□□□■■■■■■■	2 (0.18)
EAI2-nonthaburi	2848	■■□■■■■□□□□□□□□□□□□□□□□□□■□■□□□□■□■■■■■■■■■	2 (0.18)
H3	3347	□□□□□□□□□□□□□□□□□□□□□□□□■□■■■■□■□□□□■■■■■■■	2 (0.18)
T1	3505	■■■■■■■■■■■■■■■■■■■□■■■■■■■■■□■■□□□□■■■■■■■	2 (0.18)
CAS1-Kili	21	■■■□□□□■■□■■■■■■■■■□□□□□□□□□□□□□□□□■■■■■■■■	1 (0.09)
CAS1-Delhi	26	■■■□□□□■■■■■■■■■■■■■■■□□□□□□□□□□□□■■■■■■■■■	1 (0.09)
H3	36	■■■■■■■■■■■■□■■■■■■■■■■■■■■■■□■□□□□■■■■■■■	1 (0.09)
LAM7-TUR	41	■■■■■■■■■■■■■■■■■■■□□□□□■□□■■■■■□□□□■■■■■■■	1 (0.09)
Manu2	54	■■■■■■■■■■■■■■■■■■■■■■■■■■■■■■■■□□■■■■■■■■■	1 (0.09)
T5-Madrid2	58	■■■■■■■■■■■■■■■■■■■□■■□■■■■■■■■■□□□□■■■■■■■	1 (0.09)
Undefined	74	■■■■■■■■■■■■■■■■■■■■□□□□□□□□□■■■□□□□■■■■■■■	1 (0.09)
T1	86	■■■■■■■■■■■■■■■■■■■■■□■■■■■■■■■■□□□□■■■■■■■	1 (0.09)
EAI2-nonthaburi	89	■■□■■■■□□□□□□□□□□□□□□□□□□■■■□□□□■□■■■■■■■■■	1 (0.09)
T1	123	■■■■■■■■■■■■■■■■■■■■■■■■■■□□■■■■□□□□■■■■■■■	1 (0.09)
H3	134	■■■■■■■■■■■■■■■■■■■■■■■■■■■■■■□■□□□□■■□□■■■	1 (0.09)
EAI5	138	■■■■■■■■■■■■■■■■■■■■■■■■■■■■□□□□■□■■■■■□□□□	1 (0.09)
EAI4-VNM	139	■■■■■■■■■■■■■■■■■■■■■■■■■□□■□□□□■□■■■■■■■■■	1 (0.09)
T1	164	■■■■■■■■■■■■■■■■■■■■■■■□□□□□□□■■□□□□■■■■■■■	1 (0.09)
T1	169	■■■■■■■■■■■■■■■■■■■■■■■■■■■■■■■□□□□□■■■□□□□	1 (0.09)
LAM	179	■■□■■■■■■■■■□■■■■■■■□□□□■■■■■■■■□□□□■■■□□■■	1 (0.09)
X2	185	■■■■■■■■■■■■■■□■■□■■■■■■■■■■■■■■□□□□■■□□□□■	1 (0.09)
T1	191	□□■■■■■■■■■■■■■■■■■■■■■■■■■■■■■■□□□□■■■■■■■	1 (0.09)
T1	196	■■□■■■■■■■■■■■■■■■■■■■■■■■■■■■■■□□□□■■■■■■■	1 (0.09)
Undefined	237	■■■■■■■■■■■■■■■■■■■■■■■■■■■■■■□□□□□□□□□□□□□	1 (0.09)
T	240	■■■■■■■■■■■■■■■■■■■■■■■■■■■■■■■■□□□□□■■■■■■	1 (0.09)
T1	245	■■■■■■■■■■■■■■■■■■■■■■■■■■■■■■■■□□□□■■□■■■■	1 (0.09)
T5-RUS1	254	■■■■■■■■■■■■■■□□□□□□□□□□■■■■■■■■□□□□■■■■■■■	1 (0.09)
Undefined	274	■■■■■■■■■■■■■■■■■■■■□□□□□□□□□□□□□□□□□■■■■■■	1 (0.09)
T1	286	■■□■■■■■■■■■■■■■■■■□□■■■□□□□□□□□□□□□□□□□□□□	1 (0.09)
T1	306	■■■■■■■■■■■■■■■■■■■■□□□□□□■■□■■■□□□□■■■■■■■	1 (0.09)
LAM5	333	■■■■■■■■■■■■□■□■■■■■□□□□■■■■■■■■□□□□■■■■■■■	1 (0.09)
Undefined	344	■■■□□□□□□□□□■■■■■■■■■■■■■■■■■■■■□□□□■■■■■■■	1 (0.09)
X2	347	■■■■■■■■■■■■□■■■■□■■■■■■■■■■■■■■□□□□■■□□□□■	1 (0.09)
T1	373	■■■■■■■■■■■■■■■■■■■■■■■□■■■■■■■■□□□□■■■■■■■	1 (0.09)
T3	385	■■■■■■■■■■■■□■■■■□□■■■■■■■■■■■■■□□□□■□■■■■■	1 (0.09)
LAM9	388	■■■□■■■■■■■■■■■■■■■■□□□□■■■■■■■■□□□□■■■■■■■	1 (0.09)
Undefined	402	■■■■■■■■■■■■■■■■■■■■□□□□□□□□□□□□□□□□□□□□□□□	1 (0.09)
X1	408	□□■■□■■■■■■■■□■■■□■■■■■■■■■■■■■■□□□□■■■■■■■	1 (0.09)
EAI3-IND	414	■□□■■■■■■■■■■■■■■■■■■■■□■■■■□□□□■□■■□□□■■■■	1 (0.09)
T1	453	■■■■■■■■■■■■■■■■■■■■□■■■■■■■□■■■□□□□■■■■■■■	1 (0.09)
T1	455	■■■■■■■■■■■■■■■■■■■■■■■□□□□□■■■■□□□□■■■■■■■	1 (0.09)
LAM1	469	□□□■■■■■■■■■■■■■■■■■□□□□■■■■■■■■□□□□■■■■■■■	1 (0.09)
EAI2-Manila	483	■■□■■■■■■■■■■■■■■■■□□■■■■■■■□□□□■□■■■■■□□□■	1 (0.09)
LAM9	492	■■■■■■□■■■■■■■■■■■■■□□□□■■■■■■■■□□□□■■■■■■■	1 (0.09)
T	503	■■■■■■■■■■■■□■■■■■□■■■■■■■■■■■■■□□□□■■■□■■■	1 (0.09)
LAM9	509	■■■■■■■■■■■■■■■■■■■■□□□□■□■■■■■■□□□□■■■■■■■	1 (0.09)
T1	516	■■■■■■■■■■■■■■■■■■■■■■■■■■■□■■■■□□□□■■■■■■■	1 (0.09)

Undefined	563	■■■■■■■■■■■■□■■■■■■■■■■■□□□□□□□□□□□□□□□■■■■	1 (0.09)
T1	630	■■■■■■□□■■■■■■■■■■■■■□■■■■■■■■■■■■□□□□■■■■■■■	1 (0.09)
LAM	633	■■□■□■■■■■■■■■■■■■■■□□□□■■■■■■■■□□□□■■■□■■■	1 (0.09)
EAI3-IND	654	■□□■■■■■■■■■■■■■■■■■■□■■■■■■□□□□■□■■□□□■■■■	1 (0.09)
EAI6-BGD1	702	■■■□□□□□□■■■■■■■□■■■■■□□■■■■□□□□■□■■■■■■■■■	1 (0.09)
H3	714	■■■■■■■■■■■■■■■■■■□□□□□□■■□■■■□■□□□□■■■■■■■	1 (0.09)
H1	727	■■■■■■■■■■■■□■■■■■■■■■■■■□□□□□□■□□□□■■■□■■■	1 (0.09)
LAM1	729	■■□■□■■■■■■■■■■■■■■■□□□□■■■■■■■■□□□□■■■■■■■	1 (0.09)
LAM9	737	■■■■■■■■■■■■■■■■■■■■□□□□■■■■■■■■□□□□■□■■■■■	1 (0.09)
H3	740	■■■■■■■■■■■■■■■■■■■■■■□□■■■■■■□■□□□□■■■■■■■	1 (0.09)
H3	741	■■■■■■■■■■■■■■■■■■■■■■□■■■■■■■□■□□□□■■■■■■■	1 (0.09)
H3	742	■■■■■■■■■■■■■■■■■■■■■■■■□□□□□□□■□□□□■■■■■■■	1 (0.09)
LAM1	753	■□□■■■■■■■■■■■■■■■■■□□□□■■■■■■■■□□□□■■■■■■■	1 (0.09)
LAM9	822	■■■■□■■■■■■■■■■■■■■■□□□□■■■■■■■■□□□□■■■■■■■	1 (0.09)
H3	830	□□□□□□□□□□□□□□□□□□□□□□□□■■■■■■□■□□□□■■■□■■■	1 (0.09)
S	831	■■■■■■■■□□■■■■□■■■■■■■■■■■■■■■■■□□□□■■■■■■■	1 (0.09)
LAM10-CAM	838	■■■■■■■■■■■■■■■■■■■■■■□□□■■■■■■■□□□□■■■■□■■	1 (0.09)
Undefined	911	■■■■■■■■■■■■■■■■■■■■□□□□□□□□□□■■□□□□■■■■■■■	1 (0.09)
T1	926	■■■■■■□■■■■■■■■■■■■■■■■■■■■■■■■□□□□■■■■■■■	1 (0.09)
EAI5	934	■■■■■■■■■■■■■■■■■■■■■■■■■■■■□□□□■□■■□□□■■■■	1 (0.09)
Undefined	1059	■■■■■■■■■■■■■■■■■■■■■■■■■□□■□□□□■■■■■■■■■■■	1 (0.09)
H3	1085	■■■■■■■■■■■■■■■■■■■■■■■■■□□■■□□■□□□□■■■□■■■	1 (0.09)
T3	1214	■■■■■■■■■■■□□□■■■■■■■■■■■■■■■■■■□□□□■■■■■■■	1 (0.09)
T1	1233	■■■■■■■■■■■■■■■■■■□□□■■■■■■■■■■■□□□□■■■■■■■	1 (0.09)
H3	1238	■■■■■■■■■■■■■■■■■■■■■■■■■■□■■■□■□□□□■■■■■■■	1 (0.09)
X2	1270	■■■■□■■■■■■■■■■■■■□■■■■■■■■■■■■■■□□□□■■□□□□■	1 (0.09)
LAM9	1277	■■■■■■■■■■■■■■■■■■□■□□□□■■■■■■■■□□□□■■■■■■■	1 (0.09)
T1	1284	■■■■□□□□■■■■□□□□■■■■■■■■■■■■■■■■□□□□■■■■■■■	1 (0.09)
T1	1318	■□■■■■■■■■■■■■□■■■■■■■■■■■■■■■■■□□□□■■■■■■■	1 (0.09)
LAM	1321	■■□■■■■■■■■■■■■■■■■■□□□□■■■■■■■■□□□□■■■□■■■	1 (0.09)
T	1339	■■□■■■■■■■■■□■■■■■■■■■■■■■■■■■■■□□□□■■■□■■■	1 (0.09)
X3	1486	□■■□□□□□□□□□□■■■■□■■■■■■■■■■■■■■□□□□■■■■■■■	1 (0.09)
Undefined	1559	■■■■■■■■■■■■■■■■■■■□□□□□□□□□□□□□□□□□□□■■■■■	1 (0.09)
T4	1582	■■■■■■■■■■■■■■■■■■□■■■■■■■■■■■■■■□□□□■■■□□□□	1 (0.09)
H3	1611	■■■■■■■■□■■■■■■■■■■■■■■■■■■■■■□■□□□□■■■■■■■	1 (0.09)
H1	1644	■■■■■■■■■■■■■■■■■■■□□□□□■□□□□□□■□□□□■■■■■■■	1 (0.09)
T1	1645	□■■■■■■■■■■■■■□■■■■■■■■■■■■■■■■■□□□□■■■■■■■	1 (0.09)
T2	1660	■■■■■■■■■■■■■■■■■■■■□□□□□□□□□□■■□□□□■■■□■■■	1 (0.09)
T1	1761	■■□■■■■■■■■■■■□■■■■■■■■■■■■■■■■■□□□□■■■■■■■	1 (0.09)
H3	1763	■□□□■■■■■■■■■■■■■■■■■■■■■□□□■■□■□□□□■■■□■■■	1 (0.09)
AFRI_1	1782	■■■■■■□□□■■■■■■■■■■■□□□□■■■■■■■■■■■■■■□■■■■	1 (0.09)
H3	1802	■■■■■■■■■■■■■■□■■■■■■■■■■■■■■■□■□□□□■■□□■■■	1 (0.09)
H1	1807	■■■■■■■■■■■■■■■■■■■■■■■■■□□□□□□■□□□□■□■■■■■	1 (0.09)
LAM8	1815	■■□■■■■■■■■■■■■■■■■■□□□□■■□■■■■■□□□□■■■■■■■	1 (0.09)
LAM9	1832	■■■■■■■■■■■■■■■□■■■■□□□□■■■■■■■■□□□□■■■■■■■	1 (0.09)
T3	1910	■■□■■■■■■■■■□■■■■■■■■□□□■■■■■■■■□□□□■■■■■■■	1 (0.09)
T5	1912	■■■■■■■□■■■■■■□■■■■■■■□■■■■■■■■■□□□□■■■■■■■	1 (0.09)
T5	1913	■■■■■■■■■■■■■■□■■■■■■■□■■■■■■■■■□□□□■■■■■■■	1 (0.09)
LAM	2054	■■□■■■■■■■■■■■■■■■■■□□□□■■■■■■■■□□□□□■■■■■■	1 (0.09)
T1	2128	■■■■■■■■■■■■□□□■■■■■■■■■■■■■■■■■□□□□■■■■■■■	1 (0.09)
LAM9	2201	□□■■■■■■■■■■■■■■■■■■□□□□■■■■■■■■□□□□■■■■■■■	1 (0.09)
LAM5	2383	■■■■□■■■■■■■□■■■■■■■□□□□■■■■■■■■□□□□■■■■■■■	1 (0.09)
LAM3	2388	■■■■□□■■□□□■■■■■■■■■□□□□■■■■■■■■□□□□■■■■■■■	1 (0.09)
H3	2420	■■■■■■■■■■■■■■■■■■■■■■■■■■■■■■□■□□□□■■■□□□□	1 (0.09)
LAM3	2428	■■□■■■■□□□□□□□■■■■■■□□□□■■■■■■■■□□□□■■■■■■■	1 (0.09)
LAM1	2522	■■□■■■■■■■■■■■■■■■■■□□□□■■■■■■■■□□□□■■■■□□□	1 (0.09)
H3	2561	■■■■■■■■■■■■■■■■■■□□□□□□□□□□□□□■□□□□■■■■■■■	1 (0.09)
Undefined	2689	■■■■■■■■■■■■■■■■□■□□□□□□□□□□□□□□□□□□□□■■■■■	1 (0.09)
T3	2741	■■■■□■■■■■■■□■■■■■■■■■■■■■■■■■■■□□□□■■■■■■■	1 (0.09)
Undefined	2768	■■■■■■■■■■■■■■■■□□□□□□□□□□□□□□□□□□□□□□■■■■■	1 (0.09)
EAI6-BGD1	2861	■■■■■■■■■■■■■■□■□■■■■■□■■■■■□□□□■□■■□■■■■■■	1 (0.09)
EAI5	2862	■■■■■■■□□■■■■■■■■■■■■■■■■■■■□□□□■□■■■■■■■■■	1 (0.09)
LAM9	2864	■■■□□□□□□■■■■■■■■■■■■■■□□■■■□□□□■□■■■■□■■■■	1 (0.09)
LAM2	2933	■■□■■■■■■■■■□■■■■■■■□□□□■■□□■■■■□□□□■■■■■■■	1 (0.09)
LAM10-CAM	2981	■■■■■■■■□□□□□□□□□■■■■■□□□■■■■■■■□□□□■■■■■■■	1 (0.09)
Undefined	3127	□□□□□□□□□□□□□□□□□□□□□□□■■■■■■■■■□□□□■■■■■■■	1 (0.09)
T	3148	■■■■■■■■■■■■■■□■■■■■■■■■■■■■■■■■□□□□■■□□□□□	1 (0.09)
LAM3	3177	■■■■■■□□□□□□□□□□□□□■□□□□■■■■■■■■□□□□■■■■■■■	1 (0.09)
T1	3250	■■■■■■■■■■■■■■■■■□□□□■■■■■■■■■■■□□□□■■■■■■■	1 (0.09)
T2	3346	■■■■■■■■■■■■■■■■■■■■■□□□■■■■□■■■□□□□■■■□■■■	1 (0.09)
LAM1	3440	□■□■■■■■■■■■■■■■■■■■□□□□■■■■■■■■□□□□■■■■■■■	1 (0.09)
T3	3444	■■■■■■■■■■■■□■■■■■■■■■■■■■■■■■■■□□□□□□□■■■■	1 (0.09)
–	Orphan **	–	39 (3.59)

* Percentages calculated relative to the total number of drug susceptible isolates.
** Orphan profiles, i.e. profiles that were unique in the SITVIT2 database.

SUPPLEMENTAL TABLE S2: Spoligotypes obtained for *M. tuberculosis* isolates displaying antibiotic resistance (n= 153).

Lineage	SIT	Spoligotype	Nb (%)*
T1	53	■■■■■■■■■■■■■■■■■■■■■■■■■■■■■■■■□□□□■■■■■■■	21 (13.73)
LAM1	20	■■□■■■■■■■■■■■■■■■■■□□□□■■■■■■■■□□□□■■■■■■■	16 (10.46)
H2	2	□□□□□□□□□□□□□□□□□□□□□□□□■□□□□□□■□□□□■■■■■■■	8 (5.23)
Undefined	46	■■■■■■■■■■■■■■■■■■■■■■■■□□□□□□□□□□□□□□□□□□□	8 (5.23)
X3	92	■■■□□□□□□□□□■■■■■□■■■■■■■■■■■■■■□□□□■■■■■■■	7 (4.58)
X3	14	■□■□□□□□□□□□■■■■■□■■■■■■■■■■■■■■□□□□■■■■■■■	6 (3.92)
LAM6	64	■■■■■■■■■■■■■■■■■■■■□□□□■■■■□■■■□□□□■■■■■■■	6 (3.92)
H1	45	■■■■■■■■■■■■■■■■■■■■■■■□■□□□□□□■□□□□■■■■■■■	5 (3.27)
H3	50	■■■■■■■■■■■■■■■■■■■■■■■■■■■■■■□■□□□□■■■■■■■	5 (3.27)
LAM5	93	■■■■■■■■■■■■□■■■■■■■□□□□■■■■■■■■□□□□■■■■■■■	4 (2.61)
EAI6-BGD1	129	■■■□□□□□□■■■■■■■■■■■■■□□■■■■□□□□■□■■■■■■■■■	4 (2.61)
BOV_1	482	■■□■■■■■□■■■■■■□■■■■■■■■■■■■■■■■■■■■■■□□□□□	4 (2.61)
Beijing	1	□□□□□□□□□□□□□□□□□□□□□□□□□□□□□□□□□□■■■■■■■■■	3 (1.96)
T1	5	□□□□□□□□□■■□■■■■■■■■■■■■■■■■■■■■□□□□■■■■■■■	3 (1.96)
LAM9	42	■■■■■■■■■■■■■■■■■■■■□□□□■■■■■■■■□□□□■■■■■■■	3 (1.96)
T1	2791	■■■■■■■■■■■■■■■■■■■■■■■■□□□■■■■■□□□□■■■■■■■	3 (1.96)
LAM2	17	■■□■■■■■■■■■□■■■■■■■□□□□■■■■■■■■□□□□■■■■■■■	2 (1.31)
H3	75	■■■■■■■■■■■■■■□■■■■■■■■■■■■■■■□■□□□□■■■■■■■	2 (1.31)
T1	334	■□■■■■■■■■■■■■■■■■■■■■■■■■■■■■■■□□□□■■■■■■■	2 (1.31)
T3	385	■■■■■■■■■■■■□■■■■□□■■■■■■■■■■■■■□□□□■□■■■■■	2 (1.31)
EAI6-BGD1	1340	■■■■■■■■■■■■■■■■□■■■■■□■■■■■□□□□■□■■□■■■■■■	2 (1.31)
LAM9	2884	■■■■■□□□■■■■■■■■■■■■□□□□■■■■■■■■□□□□■■■■■■■	2 (1.31)
H3	3	□□□□□□□□□□□□□□□□□□□□□□□□■■■■■■□■□□□□■■■■■■■	1 (0.65)
S	34	■■■■■■■■□□■■■■■■■■■■■■■■■■■■■■■■□□□□■■■■■■■	1 (0.65)
H4	35	■■■■■■■■■■■■□■■■■■■■■■■■■■■■■□□□■□□□□■■■■■■■	1 (0.65)
T4	40	■■■■■■■■■■■■■■■■■■□■■■■■■■■■■■■■□□□□■■■■■■■	1 (0.65)
T5	44	■■■■■■■■■■■■■■■■■■■■■■□■■■■■■■■■□□□□■■■■■■■	1 (0.65)
H1	47	■■■■■■■■■■■■■■■■■■■■■■■■■□□□□□□■□□□□■■■■■■■	1 (0.65)
T1	51	■■■■■■■■■■■■■■■■■■■■■■■■■■■■■■■■□□□□■■■□□□□	1 (0.65)
T5-Madrid2	58	■■■■■■■■■■■■■■■■■■■□■■□■■■■■■■■■□□□□■■■■■■■	1 (0.65)
T1	65	■■■■■■■■■■■■■■■■■■■■■■■■■■■■■■■■□□□□■□□■■■■	1 (0.65)
X3	70	■■■□□□□□□□□□■■■■■□■■■■■■■■■■■■■■□□□□■■□■■■■	1 (0.65)
LAM6	95	■■■■■■■■■■■■■■■■■■■■□□□□■■■■□■■■□□□□■■■□■■■	1 (0.65)
T	102	■■■■■■■■■■■■□□□□■■■■■■■■■■■■■■■■□□□□■■■■■■■	1 (0.65)
X1	119	■■■■■■■■■■■■■■■■■□■■■■■■■■■■■■■■□□□□■■■■■■■	1 (0.65)
H3	134	■■■■■■■■■■■■■■■■■■■■■■■■■■■■■■□■□□□□■■□□■■■	1 (0.65)
X2	137	■■■■■■■■■■■■■■■■■□■■■■■■■■■■■■■■□□□□■■□□□□■	1 (0.65)
EAI5	152	■■■■■■■■■■■■■■■■■■■■■■■■■■□■□□□□■□■■■■■■■■■	1 (0.65)
Beijing	255	□□□□□□□□□□□□□□□□□□□□□□□□□□□□□□□□□□■■■■□■■■■	1 (0.65)
LAM5	333	■■■■■■■■■■■■□■□■■■■■□□□□■■■■■■■■□□□□■■■■■■■	1 (0.65)
Undefined	443	■■■■■■■■■■■■□■■■■■■■□□□□■■■■■■□□□□□□■■■■■■■	1 (0.65)
EAI6-BGD1	702	■■■□□□□□□■■■■■■■□■■■■■□□■■■■□□□□■□■■■■■■■■■	1 (0.65)
H3	741	■■■■■■■■■■■■■■■■■■■■■■□■■■■■■■□■□□□□■■■■■■■	1 (0.65)
Undefined	909	■■■■□□□□□■■■■■■■■■■■□■■■■■□□□□□□□□□□□□□□□■■	1 (0.65)
T	936	■■■■□□□□□■■■□■■■■■■■□■■■■■■■■■■■□□□□■■■□■■■	1 (0.65)
EAI5	1062	■■■□□□□□□■■■■■■■□□□□□□□□■■■■□□□□■□■■■■■■■■■	1 (0.65)
LAM	1086	■■■■□□□□□■■■□■■■■■■■□□□□■■■■■■■■□□□□■■■□■■■	1 (0.65)
LAM9	1241	■■■■■■■■■■■■■■■■■■■■□□□□■■■■■□□□□□□□■■■■■■■	1 (0.65)
LAM1	1935	■■□■■■■■■■■■■■□■■■■■□□□□■■■■■■■■□□□□■■□■■■■	1 (0.65)
T1	2437	■■■■■■■■■□□□■■■■■■■■■■■■■■■■■■■■□□□□■■■■■■■	1 (0.65)
–	Orphan **	–	7 (4.58)

* Percentages calculated relative to the total number of drug resistant isolates.
** Orphan profiles, i.e. profiles that were unique in the SITVIT2 database.
